# Disease features associated with a low disease impact in patients with psoriatic arthritis: results of a cross-sectional multicenter study

**DOI:** 10.1186/s13075-020-02168-1

**Published:** 2020-04-15

**Authors:** Ruben Queiro, Juan D. Cañete, María Montoro, Susana Gómez, Ana Cábez, on behalf of the MAAPS study group, Carlos Montilla, Miguel Angel Abad, Juan Carlos Torre-Alonso, Jose Andrés Román-Ivorra, Jesús Sanz, Juan Salvatierra, Jaime Calvo-Alén, Agustín Sellas, Fernando José Rodriguez, Alberto Bermúdez, Manuel Romero, Manuel Riesco, Juan Carlos Cobeta, Fermín Medina, M. Luz García, Ana Urruticoechea, Carlos González, E. Judez, Beatriz González, Pilar Fernández, Lucía Pantoja, Rosa Morlá

**Affiliations:** 1 Department of Rheumatology, HU. Central de Asturias, Avenida de Roma s/n, 33011 Oviedo, Spain; 2 Department of Rheumatology, HU. Clinic and IDIBAPS, Carrer de Villarroel, 170, 08036 Barcelona, Spain; 3 Medical Department, Pfizer, Avenida Europa 20B, Parque Empresarial La Moraleja, 28108 Madrid, Spain

**Keywords:** Psoriatic arthritis, PsAID, MDA, DAPSA, Remission, Impact of disease

## Abstract

**Background:**

Patient-reported outcomes measures, such as those provided by *the Psoriatic Arthritis Impact of Disease (*PsAID) questionnaire, have been found to be a reliable indicator of change during treatment, predictive of long-term outcomes, and the impact of psoriatic arthritis (PsA) on patients’ lives. The objective of the study was to describe the demographic and clinical characteristics of PsA patients with a low disease impact and to analyze predictive factors for that state.

**Methods:**

Post hoc analysis of a cross-sectional multicenter study that included 223 consecutive patients. PsAID questionnaire was used to estimate disease impact. Patients with a PsAID < 4 were considered in low disease impact. Minimal disease activity (MDA) response and the Health Assessment Questionnaire (HAQ) were also assessed. The degree of agreement between the different outcomes was addressed by Cohen’s kappa index.

**Results:**

One hundred and twenty-two (54.7%) patients reached a PsAID < 4. Among them, 52.0% and 68.0% presented articular or skin remission, respectively. Almost 75% of patients were in MDA state and 85.2% presented a low disability state according to the HAQ. A moderate concordance between HAQ ≤ 0.5 and PsAID < 4 (*k* = 0.53), fair between MDA and PsAID < 4 (*k* = 0.36), and moderate between DAPSA remission and PsAID < 4 (*k* = 0.46) was observed. Multivariate logistic regression analysis showed that patients with distal interphalangeal joint (DIP) disease (OR 0.40, 95%CI, 0.20–0.79, *p* = 0.009), family history of PsA (OR 0.25, 95%CI, 0.09–0.72, *p* = 0.010), and higher C-reactive protein (OR 0.92, 95%CI, 0.85–0.99, *p* = 0.036) were significantly less likely to reach a PsAID < 4.

**Conclusions:**

There is certain discrepancy between disease activity measures and a low impact of disease in PsA. Clinical features (DIP joint involvement), biologic activity, and genetic factors (familial history) seem to be associated with lower odds of reaching a low disease impact.

## Background

Patients with psoriatic arthritis (PsA) experience significant disability and reduced quality of life, resulting from the emotional distress and functional impairment associated with psoriatic skin lesions, as well as joint disease [1–3].

The Outcome Measures in Rheumatology (OMERACT) update core set for PsA evaluation comprises the domains: musculoskeletal disease activity, skin disease activity, fatigue, pain, patient global assessment (PGA), physical function, health-related quality of life (QoL), and systemic inflammation [4]. Prior literature review indicated that pain, PGA, and health assessment questionnaire (HAQ) were frequently measured; however, other measures of how the patients feel or sleep were rarely reported [5].

Gossec et al. developed and validated *the Psoriatic Arthritis Impact of Disease* (PsAID) questionnaire, which was made with extensive patient input so better relates to their concerns. PsAID can be used to calculate a score reflecting the impact of PsA on patients’ lives [6]. The PsAID questionnaire assesses 12 physical and psychological domains, ranging from pain or physical activity to anxiety or embarrassment. The PsAID is an effective QoL measure for PsA patients [3].

Patient-reported outcomes measures (PROMs), such as those provided by the PsAID questionnaire, have been found to be a reliable indicator of change during treatment, predictive of long-term outcomes, and the impact of PsA on patients’ lives [5–7]. Discrepancy between patient’s and physician’s ratings of general health status has been demonstrated [8–10]. In PsA, factors associated with discordance were psychological rather than physical and were more frequent in patients in remission [10]. The consequence of such a discordant viewpoint with regard to disease activity is that decisions are often prone to not being shared between patients and physicians [8–10]. The patients’ own perspectives of their health status may therefore be an important additional measure to assess the level of disease activity and for clinical decision-making.

The aims of the present study were to describe demographic and clinical characteristics of PsA patients with an acceptable symptoms state (PsAID score < 4), to analyze predictive factors for PsAID < 4 and the association between that state and other disease activity outcomes.

## Patients and methods

This was a post hoc analysis of data from an observational, multicenter, cross-sectional study carried out at twenty-five rheumatology outpatient clinics in the whole of Spain [11].

The study included outpatients of both genders over 18 years of age, diagnosed with PsA according to the Classification for Psoriatic Arthritis (CASPAR) criteria [12], with at least 1 year of disease duration, with hand and foot radiological tests carried out during the 6 months prior to the study visit, and under treatment with biological and/or conventional synthetic disease-modifying anti-rheumatic drugs (csDMARDs).

Data were collected between May 2014 and February 2015 at the single study visit. Patient data collection included demographics and clinical characteristics. Patients completed the self-reported PsAID questionnaire. In addition, MDA state [13] and the HAQ were assessed.

The PsAID questionnaire reflects the impact of PsA from the patients’ perspective [6]. It is comprised of 12 physical and psychological domains. Each domain is rated from 0 to 10 with a different weighting. Total score is divided by 20. The final score has a range from 0 (best status) to 10 (worst status) with a cutoff of 4. PsAID score < 4 identified a patient-acceptable symptom state (PASS), also regarded as a low disease impact [6].

Patients were considered in MDA when they met ≥ 5 of the following criteria: tender joint count ≤ 1, swollen joint count ≤ 1, Psoriasis Area and Severity Index (PASI) score ≤ 1 or body surface area ≤ 3%, patient pain visual analog scale (VAS) score ≤ 15, patient global disease activity VAS score ≤ 20, HAQ score ≤ 0.5, and tender entheseal points ≤ 1 [13].

Disease Activity in PSoriatic Arthritis (DAPSA) score was calculated by adding the number of tender and swollen joints, VAS pain, patient-reported global assessment (PGA), and C-reactive protein (CRP). The cDAPSA was calculated without the contribution of CRP. DAPSA and cDAPSA score ≤ 4 identified clinical remissions [14]. Remission was also evaluated according to the vision of the evaluating physicians (specific question yes/no).

### Statistical methodology

A descriptive statistical analysis of all the variables was performed, including central tendency and dispersion measures for continuous variables, and absolute and relative frequencies for categorical variables. Student’s *t* test, Mann-Whitney *U* test or Kruskall Wallis *H* test were used to compare quantitative variables and Pearson’s chi-square or Fisher’s exact tests for qualitative variables. Concordance was assessed using Cohen’s κappa (*k*). Univariate and multivariate models were carried out to identify factors independently associated with a PASS status. Tests were two-tailed with a significance level of 5%. Data were analyzed using SPSS V19.0 statistical software.

## Results

A total of 122 patients (54.7%) with a PsAID score < 4 (PASS) were included in the present post hoc analysis. Demographic and clinical characteristics of the study population are shown in Table 1. More than half of patients were men (57.4%) with a mean (SD) age of 54.5 (12.7) years. Mean disease duration was 9.6 (7.9) years and the musculoskeletal symptom onset occurred nearly 10 years after the skin symptoms appeared. Seventy-three patients (59.8%) were employed, while 2.5% had full disability to work due to PsA. More than half (57.4%) suffered from some kind of comorbidity. Regarding PsA condition, most patients were diagnosed with peripheral disease. Erosions in hand or feet were found in approximately 30% of patients (Table 1).

**Table 1:** Demographic and clinical characteristics of PsAID < 4 patients

	PsAID < 4, ***N*** (122)	PsAID ≥ 4, ***N*** (101)	***P*** value
Male, *n* (%)	70 (57.4)	51 (50.5)	NS
Age, mean (SD), year	54.5 (12.7)	51.7 (12.7)	NS
BMI, mean (SD) (kg/m^2^)	27.1 (3.9)	26.3 (4.8)	NS
CRP (mg/L), mean (SD)	2.8 (3.3)	4.6 (8.4)	< 0.05
Comorbidities, *n* (%)	70 (57.4)	43 (42.6)	NS
Dyslipemia	40 (32.8)	29 (28.7)	NS
HBP	33 (27.0)	27 (26.7)	NS
Obesity	30 (24.6)	16 (15.8)	NS
DM	12 (9.8)	9 (8.9)	NS
PsA clinical pattern, *n* (%)			
Axial	3 (2.5)	4 (4.0)	
Peripheral	107 (87.7)	79 (78.2)	
Mixed	12 (9.8)	18 (17.8)	
Dactylitis	64 (52.5)	47 (46.5)	NS
Enthesitis	45 (36.9)	35 (34.7)	NS
DIP	45 (36.9)	48 (47.5)	NS
Familial history, *n* (%)			
Psoriasis	60 (49.2)	51 (50.5)	NS
PsA	11 (9.0)	16 (15.8)	NS
Ankylosing spondylitis	2 (1.6)	0 (0.0)	NS
PsA duration, mean (SD), years	9.6 (7.9)	9.7 (7.7)	NS
Skin symptoms duration, mean (SD), years	21.6 (14.5)	22.9 (15.0)	NS
Articular symptoms duration, mean (SD), years	11.9 (8.7)	12.6 (10.0)	NS
Radiologic findings			
Erosions in hands, *n* (%)	40 (32.8)	43 (42.6)	NS
Erosions in feet, *n* (%)	33 (27.0)	32 (31.7)	NS

*BMI* body mass Index, *CRP* C-reactive protein, *HBP* high blood pressure, *DIP* distal interphalangeal joint disease, *DM* diabetes mellitus, *SD* standard deviation, *NS* non-significant

There were no differences in the exposure to systemic drugs among patients who achieved a PASS status compared to those who did not reach that situation. 75.4% of the subjects in the PASS state received conventional DMARDs compared to 76.2% of the non-PASS subjects. The median exposure to conventional DMARDs was 50.1 months (62.1–92.9) in the PASS subjects versus 53.9 months (52.2–84.5) in the non-PASS (*p* non-significant). With regard to biological therapies (mainly anti-TNF-alpha), 46% of PASS subjects received these agents compared to 52.5% of non-PASS (*p* non-significant). The median exposure to biologic therapy was 41.2 months (40.1–59.4) in the PASS subjects versus 35 months (31.8–50.9) in the non-PASS subjects (*p* non-significant). However, there were statistically significant differences in the exposure to systemic corticosteroids, so that 24% of non-PASS patients received these drugs compared to 12% of PASS patients, *p* = 0.025.

Clinical remission characteristics of the study population are shown in Table 2. According to physician criteria, 52.0% and 68.0% of patients presented articular or skin remission, respectively. MDA response was achieved by 74.5% of patients in PASS state. The most common MDA active domains were *patient pain VAS ≤ 1.5* and *global disease activity VAS score ≤ 20* achieved only by 53.4% and 55.7% of PsAID < 4 patients, respectively (Table 2). The majority (85.2%) of patients presented a low disability state according to HAQ [mean (SD) total score 0.2 (0.3)].

**Table 2:** Clinical remission characteristics of PsAID < 4 patients

	PsAID < 4	PsAID ≥ 4	***P*** value
Articular remission, *n* (%)*	53 (52.0)	23 (23.5)	< 0.001
Skin remission, *n* (%)*	83 (68.0)	51 (50.5)	< 0.01
MDA, *n* (%)			
Tender joint count ≤1, *n* (%)	91 (74.6)	53 (52.5)	< 0.001
Swollen joint count ≤1, *n* (%)	82 (67.2)	70 (69.3)	NS
PASI ≤ 1/Body surface area ≤ 3%, *n* (%)	96 (79.3)	78 (78.8)	NS
Patient pain VAS ≤ 1.5, *n* (%)	63 (53.4)	7 (7.1)	< 0.0001
Patient global disease activity VAS ≤ 20, *n* (%)	68 (55.7)	28 (27.7)	< 0.0001
HAQ ≤ 0.5, *n* (%)	104 (85.2)	33 (32.7)	< 0.0001
Tender entheseal points ≤ 1	99 (81.8)	80 (79.2)	NS
PASI, mean (SD)	1.2 (3.8)	1.8 (3.2)	NS
HAQ, mean (SD)	0.2 (0.3)	0.9 (0.6)	< 0.001

*According to physicians criteria

*MDA* minimal disease activity, *PASI* Psoriasis Area and Severity Index, *VAS* visual analog scale, *HAQ* Health Assessment Questionnaire, *PsAID* Psoriatic Arthritis Impact of Disease, *CI* confidence intervals

A moderate concordance between HAQ ≤ 0.5 and PsAID < 4 (kappa [CI_95%_] 0.53 [0.42–0.64]) and fair between MDA and PsAID < 4 (kappa [CI_95%_] 0.3594 [0.2393–0.4795]) was observed. Concordance between PsAID < 4 and skin (kappa [CI_95%_] 0.28 [0.15–0.41]) or articular (kappa [CI_95%_] 0.18 [0.05–0.31]) remission was considered poor. Kappa agreement between DAPSA remission and PsAID < 4 was 0.46 (CI_95%_ 0.34–0.58), (moderate agreement), while kappa value between cDAPSA remission and PsAID < 4 was 0.58 (CI_95%_ 0.47–0.70) (moderate agreement).

The variables associated with the PASS status in the univariate analysis (*p* < 0.10) were age (OR 1.02), secondary education (OR 0.45), active workers (OR 2.23), retired (OR 2.72), smoking (OR 0.46), peripheral disease (OR 2.03), DIP involvement (OR 0.64), PsA family history (OR 0.52), coronary heart disease (OR 7.02), obesity (OR 1.73), CRP (OR 0.94), glucocorticoids (OR 0.45), and syndesmophytes (OR 0.44). Multivariate logistic regression analysis showed that patients with DIP joint involvement (odds ratio (OR) [95%CI], 0.40 [0.20–0.80]; *p* = 0.009), family history of PsA (OR 0.25 [0.09–0.72]; *p* = 0.010), and high CRP (OR 0.92 [0.85–0.99]; *p* = 0.036), were significantly less likely to reach a PsAID score < 4.

## Discussion

In this post hoc analysis of the MAAPs study, we verified that a high percentage (54.7%) of patients with established PsA undergoing systemic therapies reached a low disease impact according to the PsAID questionnaire. However, joint or skin remission, defined by the evaluating physician, was not consistent with an acceptable symptomatic status. The agreement between other disease outcomes, such as the MDA response, or the HAQ, was fair or moderate, with respect to the PASS state. On the other hand, the presence of DIP joint involvement, a higher CR*P* value at study visit, or a family history of PsA, diminished the odds of having reached a low impact of disease according to the PsAID.

In recent years, the rheumatology community has witnessed an intense search for sensitive and valid instruments that capture those clinical aspects related to disease activity, as well as those linked to the response to the scheduled treatments, along with other instruments, more focused on the experiences and perceptions lived by PsA patients [3–6, 15]. Despite this intense search, there is a remarkable mismatch between the results derived from the instruments for measuring disease activity (e.g., DAPSA), or those that assess the treatment objectives (e.g., MDA), and the PROMs (e.g., pain, fatigue, PsAID). Our results do not escape this decoupling, and in fact, the worst agreement was between the definition of remission by clinicians, and the PASS state according to the PsAID questionnaire. On the contrary, the best agreement was seen between cDAPSA remission and PASS. In this sense, our findings are quite consistent with those of the recently published ReFlap (Remission/Flare in PsA) study [16]. In ReFlap, adults with physician-confirmed PsA and > 2 years of disease duration in 14 countries were included. Remission was defined as *very low disease activity* (VLDA), DAPSA ≤ 4, and physician-perceived and patient-perceived remission. In this study, DAPSA-based remission/low disease activity (LDA) performed better than VLDA/MDA to detect patient defined remission or remission/LDA. Further, physician-perceived remission/LDA using a single question was frequent (67.6%) but performed poorly against other definitions [16].

Which are the drivers of this discordance between patient-perceived remission, or low impact of disease, and physician-defined remission? Discordance between patients and physicians may vary according to levels of disease activity. Possibly, in high disease activity, both patient and physician global assessments (PGA/PhGA) will be unsatisfactory, whereas in LDA, patient and physician expectations may differ more frequently. In a recent study that included 460 patients (40.4% undergoing treatment with biologic agents), discordance defined by |PGA − PhGA| ≥ 3 of 10, concerned 134 patients (29.1%), and 115 patients (85.8% of the patients with discordance) had PGA > PhGA. Higher fatigue, lower self-perceived coping, and impaired social participation were independently associated with a higher difference PGA − PhGA [10]. Authors concluded that factors associated with discordance were psychological rather than physical domains of health. In our study, the most active components of the MDA response in subjects with high disease impact were pain, PGA, and HAQ ≥ 0.5. This may reflect that pain (regardless of inflammatory activity) and physical dysfunction are the main drivers of a state of high disease impact in these patients. In short, our findings are in line with previous studies that indicate a clear discrepancy between the results of the activity and impact outcomes of the disease represented in such indices as the PsAID. An instrument like the PsAID, which integrates not only physical but also psychosociological domains, certainly comprises aspects that are not included in responses such as the MDA or the DAPSA score, not even indirectly. It is therefore to be expected that concordance between the results of both tools only be moderate, as we observe in this study, both for MDA/PsAID and for DAPSA/PsAID.

This mismatch between the visions of doctors and patients is not a minor matter [17]. In another recent study, it was found that one third of patients in a clinically acceptable condition according to the evaluating physician did not reach the MDA response, so that if a treat to target strategy had been applied, these patients should have received a therapeutic intensification [18]. Therefore, we need to balance the information from conventional activity measures, with those reflecting patient’s perceptions, in order to make clinical and therapeutic decision-making that conform to current disease management recommendations [19].

The nail plates and DIP joints are closely connected through a network of entheses, so that inflammation at these anchor points is the common immune connector to both domains of psoriatic disease [20]. Although the involvement of DIP joints is a well-known feature of PsA, little is known about the effect of this manifestation on QoL, the impact of the disease, or the achievement of treatment goals. A very recent study showed that DIP disease decreased the odds of having reached the MDA response [21]. In our study, we have found a negative association between this manifestation and the PASS state; however, we did not detect any link between this trait and the MDA response. We can speculate on the fact that perhaps some of these patients could actually have DIP osteoarthritis (OA), which is usually associated with OA at other locations, resulting in a worse functional status. However, one of the exclusion criteria of this study was the presence of a suspected or confirmed hand OA.

We also found that a positive PsA family history decreased the chances of reaching a state of low disease impact. In a classic study, HLA antigens B27, when DR7 was present, and DQw3, when DR7 was absent, predicted disease progression across all transitions, while HLA-B39 was associated with progression in early disease [22]. Disease progression, understood as progression of structural damage, is the main factor associated with a high HAQ (one of the most active components in our patients with high disease impact) [23]. This may allow us to speculate with some genetic background driving the factors associated to the perception of disease impact. However, the concept of disease impact is multifactorial, so this assumption does not go beyond being a mere hypothesis that requires adequately designed studies.

A recent subanalysis of the ReFlap study showed that high life impact (PsAID ≥ 4) was associated with female gender, enthesitis, tender joints, and comorbidities [24]. Although in our study more men reached an MDA status, none of the factors found in the aforementioned Reflap study was associated with a high impact of disease in our population. Even more, some comorbidities (obesity, CHD) were positively associated with a low impact of disease in our series. Although obesity, and other elements of the metabolic syndrome, has been associated with worse QoL and worse therapeutic response in PsA, not all studies point in that direction [25, 26].

Our study has the limitations of any cross-sectional study. It is, therefore, an observation at a specific timepoint of a disease, such as PsA, which tends to a high phenotypic variability over time. In addition, the instrument chosen to measure disease activity (e.g., DAPSA) does not include important aspects of the disease (axial involvement, skin, enthesitis). On the other hand, the PsAID questionnaire surely captures many patient’s perceptions, which go far beyond what can be included in a therapeutic objective such as MDA. Therefore, mismatch between these instruments is not unexpected.

## Conclusions

The impact that PsA generates on patients’ must be adequately addressed. In PsA, certain enthesitis-related characteristics (DIP disease), a high biologic activity (CRP), and some ill-defined genetic factors (family history) may contribute to a higher disease impact perception. This knowledge may help rheumatologists to better manage this condition.

## Data Availability

The datasets used and/or analyzed during the current study are available from the corresponding author on reasonable request.

## Abbreviations

CASPAR: Classification for Psoriatic Arthritis
CRP: C-reactive protein
DIP: Distal interphalangeal joint
DMARDs: Disease-modifying anti-rheumatic drugs
HAQ: Health Assessment Questionnaire
MDA: Minimal disease activity
OMERACT: Outcome Measures in Rheumatology
PASI: Psoriasis Area and Severity Index
PGA: Patient’s global assessment
PROMs: Patient-reported outcomes measures
PsAID: Psoriatic Arthritis Impact of Disease
PsA: Psoriatic arthritis
QoL: Quality of life
VAS: Visual analog scale
